# Dynamic modulation of social influence by indirect reciprocity

**DOI:** 10.1038/s41598-021-90656-y

**Published:** 2021-05-27

**Authors:** Joshua Zonca, Anna Folsø, Alessandra Sciutti

**Affiliations:** 1 Cognitive Architecture for Collaborative Technologies (CONTACT) Unit, Italian Institute of Technology, Via Enrico Melen, 83, 16152 Genoa, GE Italy; 2 Department of Informatics, Bioengineering, Robotics and Systems Engineering, University of Genoa, Genoa, Italy

**Keywords:** Human behaviour, Social behaviour, Decision, Morality

## Abstract

Indirect reciprocity is a pervasive social norm that promotes human cooperation. Helping someone establishes a good reputation, increasing the probability of receiving help from others. Here we hypothesize that indirect reciprocity regulates not only cooperative behavior but also the exchange of opinions within a social group. In a novel interactive perceptual task (Experiment 1), we show that participants relied more on the judgments of an alleged human partner when a second alleged peer had been endorsing participants’ opinions. By doing so, participants did not take into account the reliability of their partners’ judgments and did not maximize behavioral accuracy and monetary reward. This effect declined when participants did not expect future interactions with their partners, suggesting the emergence of downstream mechanisms of reciprocity linked to the management of reputation. Importantly, all these effects disappeared when participants knew that the partners’ responses were computer-generated (Experiment 2). Our results suggest that, within a social group, individuals may weight others’ opinions through indirect reciprocity, highlighting the emergence of normative distortions in the process of information transmission among humans.

## Introduction

**Figure 1:**
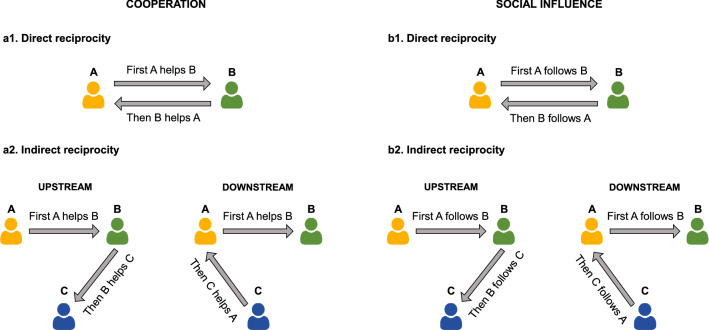
Schematic representation of direct and indirect reciprocity in cooperation (**a1**,**a2**) and social influence (**b1**, **b2**) scenarios. (**a1**) Direct reciprocity in cooperation. An agent A benefits an agent B and then B benefits A. (**a2**) Indirect reciprocity in cooperation. In upstream indirect reciprocity, an agent A benefits an agent B and then B benefits a third agent C. In downstream indirect reciprocity, A benefits B and then C benefits A. (**b1**) Direct reciprocity in social influence. An agent A relies on the opinions of an agent B and then B relies on the opinions of A. (**b2**) Indirect reciprocity in social influence. In upstream indirect reciprocity, an agent A relies on the opinions of an agent B and then B relies on the opinions of a third agent C. In downstream indirect reciprocity, A relies on to the opinions of B and then C relies on the opinions of A.

Cooperation is one of the hallmarks of human behavior. The question of how natural selection has favored cooperation in humans has fascinated scientists for decades. Indeed, cooperation needs effective mechanisms to resist exploitation arising from defective behavior, which generally provides immediate benefits for defectors. One of these mechanisms is definitely reciprocity, which assumes that one’s tendency to cooperate is conditional upon cooperative behavior of others^1,2^. Reciprocity can sustain the evolution and the maintenance of cooperation within groups^3,4^ and has been established in human societies as a social norm^5–7^. Reciprocity can arise in two main forms: direct and indirect (Fig. 1a1,a2). In *direct* reciprocity, individuals return the help received in repeated encounters with the same partner. *Indirect* reciprocity generalizes to environments where interactions may occur with unknown partners^8–10^. In particular, indirect reciprocity can emerge through two different mechanisms (Fig. 1a2). First, individuals who have recently received help may feel the urge to help someone else, a mechanism referred to as *upstream* indirect reciprocity^11–13^, which has been shown to be supported by the sentiment of gratitude^14^. Indirect reciprocity may also arise in the form of *downstream* indirect reciprocity, which predicts that helping others increases the probability of receiving help by modulating reputation^3,5,15–18^.

In all these forms of reciprocity, the recipient of an act of altruism decides to pay an individual cost to benefit the donor (direct reciprocity) or someone else (indirect reciprocity), fueling the social mechanisms that promote and sustain cooperation in humans. Although reciprocity has historically been studied in settings involving cooperation, we highlight that the normative mechanisms underlying reciprocal behavioral emerge in other types of social scenarios. In particular, extensive evidence has shown that social norms modulate behavior in contexts of social influence, where individuals exchange information and can use others’ opinions to modify their own judgments^19,20^ and preferences^21,22^. For instance, people tend to normatively conform to their peers’ opinions in order to affiliate with them and increase self-image^19,23–25^, even when this represents a behavioral cost (i.e., decreases behavioral accuracy). In this regard, recent evidence by Mahmoodi and colleagues^26^ has revealed that *direct* reciprocity modulates social influence and advice taking in repeated exchange of information between *dyads*. In particular, people are more susceptible to the advice of a partner if the partner has previously assigned high weight to their own opinions, independently of their actual belief about the partner’s reliability as information source. The authors have suggested that this behavior is consistent with a process of norm abiding, which leads individuals to pay a cost (by not optimizing behavioral accuracy) to adhere to a social norm and maintain influence over their partner. This interpretation is grounded on two assumptions: (1) exerting influence over others is rewarding^27^, whereas being ignored is perceived as distressful and painful^28^; (2) taking into account the opinions of others (and not ignoring them) is therefore perceived as a social norm^26^. In these terms, reciprocity supports the maintenance of a pro-social attitude among interacting partners who are willing to sacrifice behavioral accuracy (by showing consideration for others’ opinions) in order to exert influence over them.

Nevertheless, no study to date has investigated whether *indirect* reciprocity can modulate social influence in *multi-agent* scenarios. In the current study, we hypothesize that individuals’ susceptibility to the opinion of a person can be modulated not only by the susceptibility of that same person (direct reciprocity of social influence, Fig. 1b1) but also by the level of susceptibility of other peers within the same social group (indirect reciprocity of social influence, Fig. 1b2). In this scenario, reciprocal social influence would act as a group norm that evolves and consolidates within a social group without the need of repeated encounters between the two same individuals. Indirect reciprocity would therefore provide a model describing how the exchange of information between the members of a social group can be distorted by the emergence of group norms and by the strategic control of reputation.

In order to test this hypothesis, we designed an experimental paradigm (Experiment 1) inspired by previous work on *direct* reciprocity in social influence^26^. Our experimental paradigm includes two tasks: *Social influence* and *Indirect reciprocity*. In the *Social influence task*, we tested the participants’ willingness to rely on the opinion of an alleged partner in a perceptual inference task, in light of participants’ perceived reliability of the partner’s estimate. In the *Indirect reciprocity task*, we investigated whether participants’ willingness to take into account their partner’s perceptual estimates could dynamically change as a function of the level of susceptibility that a second alleged partner, in turn, expressed towards participants. Participants were incentivized to be as accurate as possible in their perceptual judgments. Nevertheless, participants knew that all their responses would be observed by their partners (and vice versa), which allowed us to explore the emergence of potential *downstream* effects of indirect reciprocity based on within-group reputation management (Fig. 1b2). We predict that participants would be more influenced by the judgments of a peer if someone else has previously put high weight on participants’ ones. Moreover, we hypothesize that this effect is supported by downstream reciprocity: in particular, we expect that participants would rely less on reciprocal behavior if they do not expect future interactions with their partners.

These hypotheses assume that reciprocal behavior is grounded on normative mechanisms regulating social influence among peers. In other words, reciprocity should not emerge if we remove the social component from the multi-agent interactive scenario. Therefore, we ran an additional control experiment (Experiment 2) to ensure that the effects of indirect reciprocity of social influence observed in Experiment 1 were indeed due to social phenomena. A new pool of participants performed the same tasks of Experiment 1, but this time we did not provide any cover story to participants, who were simply told that the partners’ responses in both tasks had been generated by computer algorithms. Any other aspects of the task were identical to those in Experiment 1. In this case, we do not expect any effect of indirect reciprocity in terms of participants’ susceptibility to the computer judgments, which should be exclusively weighted by their reliability.

## Results

### Experiment 1

#### Social influence task

**Figure 2:**
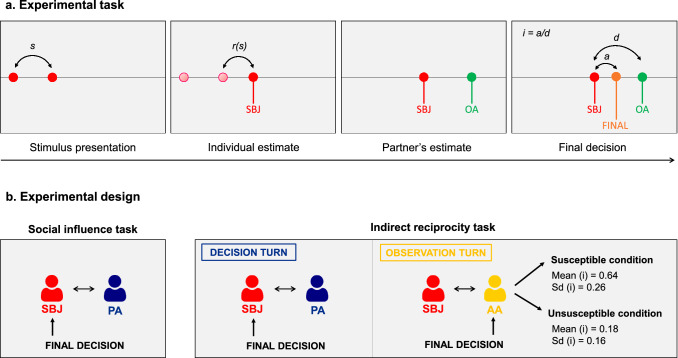
(**a**) Experimental task. In both the *Social influence* and the *Indirect reciprocity* task, initially participants had to produce perceptual estimates by reproducing the length of visual stimuli. They saw two consecutive light flashes (i.e., red disks) and had to touch a point, to the right of the second disk, in order to reproduce the stimulus length (s), defined as the distance between the first and the second disk. Participants were told that the very same stimuli would be presented to an alleged partner (other agent: OA) who would choose a point to reproduce the same distance. After the participants’ estimate, the other agent’s simulated estimate was shown, and then one of the two agents had the opportunity to make a final decision by choosing any position between their previous response and the other agent’s response. We computed participants’ influence (i) as the adjustment towards the partner in the final decision (a) divided by the distance between the two agents’ responses (d). (**b**) Experimental design. The experimental paradigm consisted of two main tasks: *Social influence *and* Indirect reciprocity*. Both tasks were based on the perceptual inference task described above, but differed in the type of interaction with the partner(s) and the identity of the agent taking the final decision. In the *Social influence task*, participants interacted with an alleged partner (passive agent: PA) and had to personally make final decisions (i.e. revising their estimates in light of the passive agent’s one) in all trials. In the *Indirect reciprocity task*, in half of the trials (decision turns) participants performed the same task with the passive agent and always made the final decision. Decision turns were alternated with observation turns, in which participants interacted with another alleged agent (active agent: AA). In these trials, the two agents made their perceptual estimates, and then the active agent took the final decision, while participants could not revise their estimate. We manipulated the active agent’s final decisions to express two different levels of susceptibility towards participants’ responses (Susceptible and Unsusceptible conditions).

In the *Social Influence task* (Fig. 2), 36 participants performed 66 trials of an interactive perceptual task with an alleged participant (passive agent). In each trial, participants estimated the length of a visual stimulus and then saw the estimate made by the passive agent concerning the same stimulus. Participants did not receive any feedback on the accuracy of the two responses. Afterwards, participants could revise their estimate by allocating a final response in any position between their own and the passive agent’s initial estimate. We computed the *influence* that the partner exerted on participants as the distance between the participants’ final and initial response divided by the distance between the two agents’ initial estimates (Fig. 2a). Therefore, *influence* is a continuous measure that can assume any value between 0 and 1, where 0 indicates a final decision coinciding with the participant’s initial estimate and 1 corresponds to a final decision coinciding with the partner’s estimate. In this way, our index of influence expresses the relative weight assigned to the judgment of each of the two interacting partners. Participants were told that their reimbursement would be calculated based on the accuracy of both their own initial estimate and their final decision. Participants were also told that the partner could see both their initial and final estimates.

**Figure 3:**
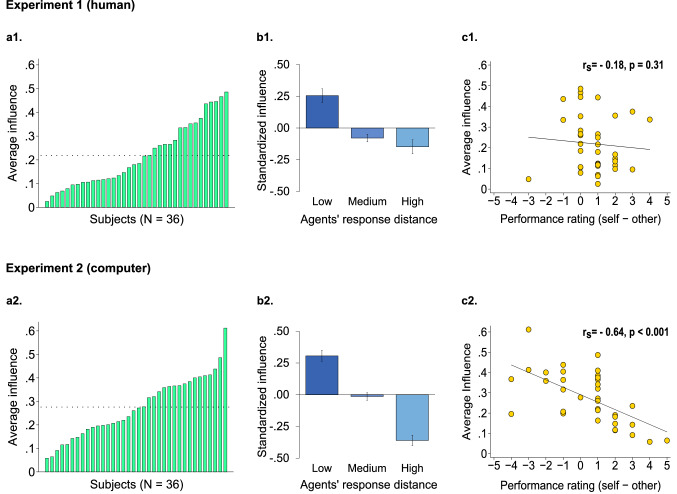
Results of the *Social influence task* in Experiment 1 (**a1**,**b1**,**c1**) and Experiment 2 (**a2**,**b2**,**c2**). In Experiment 1, participants believed to be interacting with a human partner, while in Experiment 2 they were aware of interacting with a computer. The partner’s responses were identical across experiments. (**a1**,**a2**) Average influence (i.e., shift towards the partner’s response in the final decision) plotted across participants in Experiment 1 and Experiment 2, respectively. (**b1**,**b2)** Standardized influence plotted as a function of agents’ response distance. To obtain this graph, for each participant, we standardized trial-by-trial normalized distance between the estimates of the two agents [distance (cm)/stimulus length (cm)] to express each distance value in terms of deviation from the individual subject mean. We clustered standardized distances in three bins: low (distance < − 0.5 SD); medium (− 0.5 SD ≥ distance < 0.5 SD); high (distance ≥ 0.5 SD). We also standardized, for each participant, trial-by-trial influence to express influence values in terms of deviation from individual influence means. Eventually, for each distance range, we averaged individual influence means across subjects. Error bars represent between-subject standard errors. (**c1**,**c2**) Scatter plot of individual average influence as a function of the difference between self and partner rating of perceptual inference accuracy, assessed at the end of the Social influence task in both experiments. The two measures are not correlated in Experiment 1, whereas we found a strong correlation in Experiment 2.

Although participants showed marked heterogeneity in terms of influence, they all relied more on their own initial response (Mean influence ± std. dev. = 0.22 ± 0.14, Fig. 3a1).

Then we tested whether participants’ influence was predicted by the distance from the partner’s response (normalized by stimulus length), which can modulate the attribution of competence to the two agents. A mixed-effect model revealed that within-subject heterogeneity in influence was indeed predicted by the two agents’ response distance (unstandardized coefficient (B) = − 0.181, standardized coefficient (β) = − 0.145, z = − 3.28, *p* = 0.001, Supplementary information, model 1.1). In particular, participants’ influence decreased as the distance between the two agents’ responses increased, suggesting that participants interpreted higher discrepancies between the two agents’ responses as a signal of the unreliability of the partner (Fig. 3b1). To corroborate this hypothesis, we analyzed participants’ ratings of their own and partner’s accuracy (from 1 to 10), which were collected at the end of the task. We found that participant’s ratings of their own accuracy were indeed significantly higher than ratings of the partner’s accuracy (own: 6.25 ± 1.23; partner: 5.44 ± 1.32. Wilcoxon signed-rank test, z = 3.57, r = 0.42, η^2^ = 0.18, *p* < 0.001). This is particularly interesting if we consider that participants’ accuracy in terms of perceptual estimates was markedly lower than that of the partner (perceptual error (cm) normalized by stimulus length (cm), participants: 0.22 ± 0.08; partner: 0.10 ± 0.01. Wilcoxon rank-sum test, z = − 6.96, r = 0.82, η^2^ = 0.67, *p* < 0.001). Participants’ estimation error was a poorer predictor of within-subject variability in influence than agents’ response distance (Mixed-effect model, B = − 0.100, β = − 0.075, z = − 2.06, *p* = 0.039, Supplementary information, model 1.2), although the two measures are highly correlated (Spearman correlation, rho = 0.71, *p* < 0.001). For this reason, we used agents’ response distance as control variable for subsequent analyses in the *Indirect reciprocity task*.

Although individuals’ trial-by-trial modulation of influence depended on the current distance from the partner’s response, we did not find any between-subject correlation between participants’ average influence and individual predictors such as mean normalized distance between the two agents’ estimates (Spearman correlation, rho = − 0.11, *p* = 0.519), mean normalized estimation error (rho = − 0.18, *p* = 0.302), ratings of own (rho = 0.01, *p* = 0.969) and passive agent’s (rho = 0.11, *p* = 0.535) accuracy, as well as the difference between ratings of own and passive agent’s accuracy (rho = − 0.18, *p* = 0.307, Fig. 3c1). These null results suggest that between-subject heterogeneity in susceptibility was not simply driven by differences in the perceived reliability of the partner’s estimates.

#### Indirect reciprocity task

Right after the *Social influence task*, the same participants underwent the *Indirect reciprocity task* (Fig. 2). Half of the trials (decision turns) were identical to those of the *Social influence task* and consisted of an interaction between the participant and the previous alleged partner (passive agent): the participant produced a perceptual estimate, then observed the passive agent’s estimate and eventually made a final decision. Decision turns were continuously alternated with observation turns, in which participants interacted with another alleged participant (active agent); in these trials, the participant and the active agent produced their perceptual estimates, and the latter made the final decision, revising their own estimate in light of that of the participant. In observation turns, we systematically manipulated how much the active agent was influenced by the participant in their own final decisions. In the Susceptible condition, the active agent revised their estimate considerably (average influence = 0.64, mean within-subject standard deviation = 0.26), whereas in the Unsusceptible condition they were much less influenced by the participant’s opinion (average influence = 0.18, mean within-subject standard deviation = 0.16). Participants were informed that each of the three agents could observe perceptual estimates and final decisions of the other peers in every turn. They were also told that their reimbursement would be determined by the accuracy of both their *own* perceptual estimates and *own* final decisions, as in the *Social influence task*. In this way, participants were incentivized to be as accurate as possible, independently of the partners’ behavior. We hypothesize that the amount of advice taken from the participant by the active agent in the observation turns can modulate how susceptible participants will be toward the passive agent in the decision turns, following indirect reciprocity mechanisms. Ratings of own and partners’ accuracy were collected during Susceptible and Unsusceptible conditions (See the “Methods” section for details).

**Figure 4:**
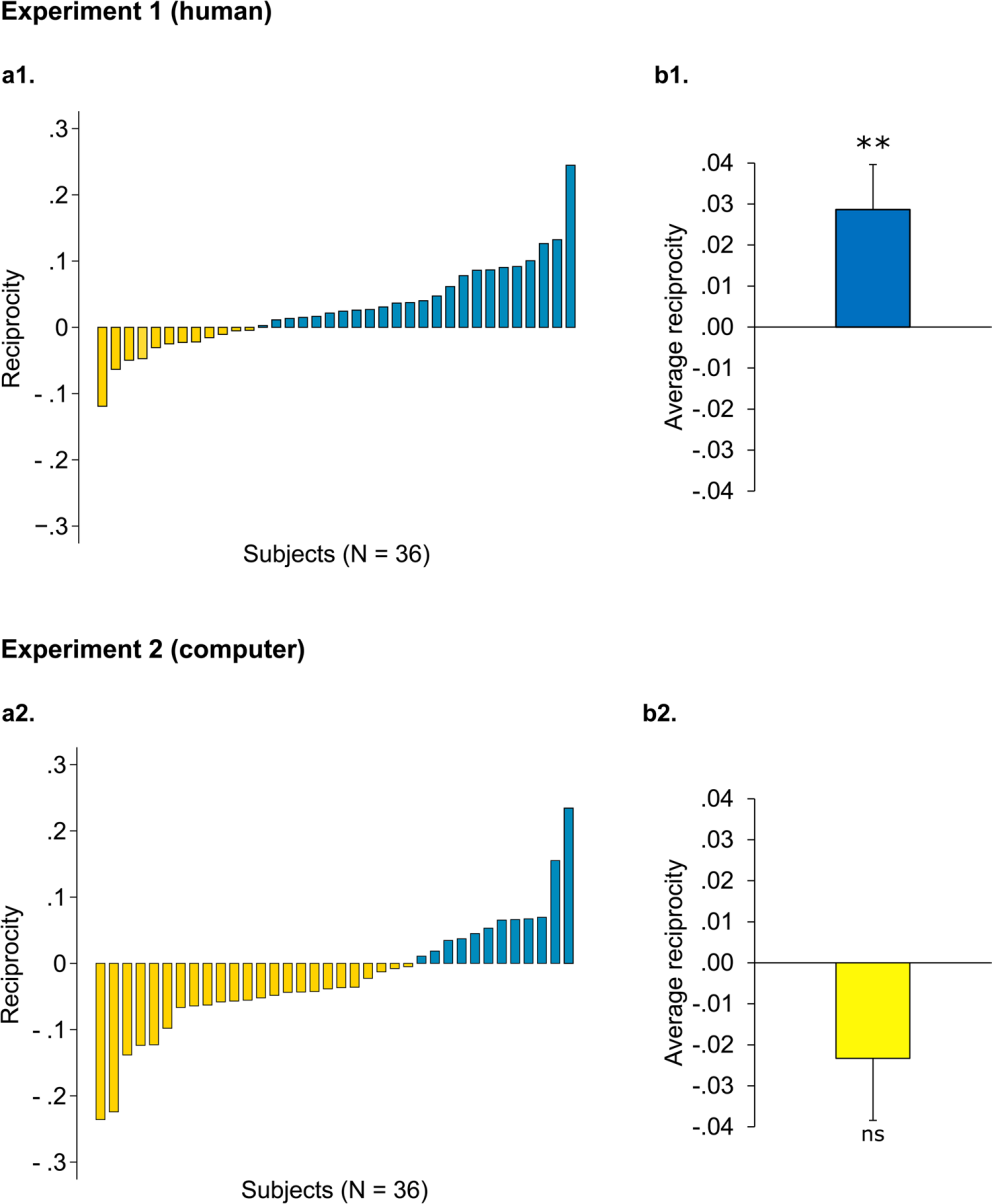
Results of the *Indirect Reciprocity task* in Experiment 1 (**a1**,**b1**) and Experiment 2 (**a2**,**b2**). (**a1**,**a2**) Reciprocity index, computed as mean influence in the Susceptible condition minus mean influence in the Insusceptible condition (in decision turns), plotted across participants. Negative values (in gold) represent participants who show anti-reciprocal behavior, while positive values (in blue) represent individuals who show indirect reciprocity and therefore increased the level of influence taken from the passive agent in the Susceptible condition. (**b1**,**b2**) Average reciprocity index in Experiment 1 and Experiment 2. Error bars represent between-subject standard error of the mean. **p < 0.01, ns: not significant. Effect of condition in Mixed-effect model.

In order to compare participants’ influence in the two conditions, we ran a mixed-effect model with influence (in decision turns) as dependent variable and condition (Susceptible or Insusceptible) as factor (Supplementary information, model 1.3). Results show a significant effect of condition (B = 0.029, β = − 0.122, z = 2.61, *p* = 0.009), revealing that influence was higher in the Susceptible than in the Unsusceptible condition (Susceptible: 0.29 ± 0.15; Unsusceptible: 0.26 ± 0.13. Figure 4.a1, 4.b1). The observed effect of indirect reciprocity was confirmed by a Wilcoxon signed-rank test revealing a significant difference in influence across conditions (z = 2.47, r = 0.29, η^2^ = 0.09, *p* = 0.014). This effect held when controlling for the distance from the participant’s and the passive agent’s estimate (Supplementary information, model 1.4).

The observed increase in influence in the Susceptible condition cannot be explained by a change in the perception of the passive agent’s accuracy, since ratings of the passive agent’s accuracy were very similar across conditions (Susceptible: 5.94 ± 1.17; Unsusceptible: 5.94 ± 1.39. Wilcoxon signed-rank, z = − 0.07, r = 0.01, η^2^ = 0.00, *p* = 0.941). Moreover, we observe that own performance ratings were higher in the Susceptible condition (Susceptible: 6.78 ± 1.20; Unsusceptible: 6.5 ± 1.32.Wilcoxon signed-rank: z = 2.14, r = 0.25, η^2^ = 0.06, *p* = 0.032), suggesting that participants, in the Susceptible condition, took more into account the opinion of the passive agent *although* they felt more confident about their own accuracy. This is not consistent with a process of information weighting based on reliability: in that case, we should expect a *decrease* in participants’ susceptibility to others’ opinions whenever participants feel more confident about their own judgements.

**Figure 5:**
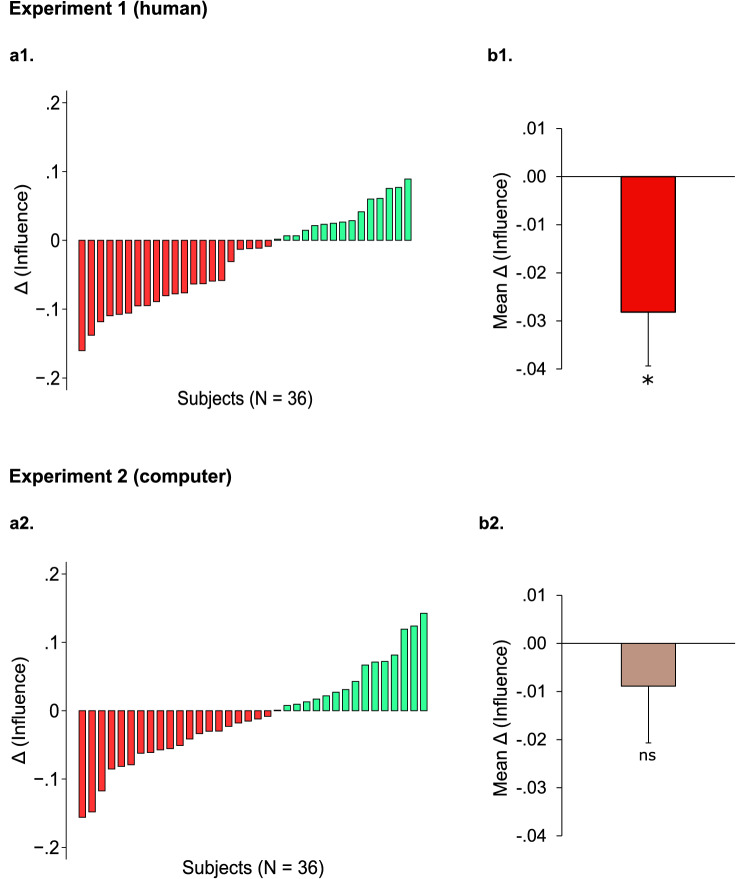
Effect of Final block in the *Indirect reciprocity task* in Experiment 1 (**a1**,**b1**) and Experiment 2 (**a2**,**b2**). (**a1**,**a2)** Δ (Influence) in the Final block, computed as mean influence in the Final block minus mean influence in the previous main block of the Indirect reciprocity task, plotted across participants. Negative values (in red) represent participants who decreased the amount of advice taken from the passive agent in the Final block, while positive values (in green) represent individuals who increased their level of susceptibility towards the passive agent in the Final block. (**b1**,**b2)** Mean Δ (Influence) in the Final block in Experiment 1 and Experiment 2. Error bars represent between-subject standard error of the mean. *p < 0.05, ns: not significant. Effect of block in Mixed-effect model.

Eventually, we tested the hypothesis that the emergence of reciprocal social influence in participants is sustained by the expectation of future interactions with their partners, following mechanisms of downstream reciprocity based on reputation management. If this is the case, we should observe a drop in terms of influence if participants do not expect future interaction with the active agent. To test this hypothesis, at the end of the experiment we added an experimental block (Final block), characterized by a different alternation of turns: first participants faced 11 observation turns in a row (Susceptible or Unsusceptible, consistently with the last main block) and eventually performed 11 decision turns in a row. Participants were informed about this change in the order of the turns only at the beginning of the Final block and were told that the experiment be finished after the 11 decision turns. Nonetheless, participants were told that their choices would be observed by their partners. We compared influence in the Final block with influence in the preceding main experimental block: these two blocks indeed coincide in terms of behavior of the active agent, but differ in the prospect of future interactions with the active partner. Results of a mixed-effect model (Supplementary information, model 1.5) show a significant drop in participants’ influence in the Final block (B = − 0.028, β = − 0.119, z = − 2.52, *p* = 0.012, Fig. 5a1,b1). We found the same effect when adjusting for effects of distance between agents’ responses (Supplementary information, model 1.6). The observed effect of influence decrease in the Final block was corroborated by a Wilcoxon signed-rank test revealing a significant difference in influence across blocks (z = − 2.17, r = 0.26, η^2^ = 0.07, *p* = 0.030). These results suggest that expectation of future interactions within a group supports downstream indirect reciprocity of social influence.

### Experiment 2

In Experiment 2, we tested a new pool of 36 participants in the *Social influence task* and the *Indirect reciprocity task*, but this time we did not provide any cover story to participants. More specifically, participants knew that they were performing the two tasks with computer algorithms rather than real participants. Experiment 2 will serve as a control experiment for Experiment 1. Since we believe that the effects shown in Experiment 1 depend on social and normative mechanisms induced by the cover story, we do not expect any effect of reciprocity in Experiment 2, where the computer judgments should be weighted exclusively by their reliability.

#### Social influence task

In Experiment 2, a new pool of participants performed the *Social influence task* with the same modalities of participants in Experiment 1, but they were aware that their partner was in fact a computer algorithm and not a human participant. Nonetheless, the pattern of responses generated by the algorithm was identical in the two experiments. Participants did not receive any information about the response patterns of the algorithm.

In line with results of Experiment 1, participants who knew that their opponent was a computer algorithm relied more on their own response (Mean influence ± std. dev. = 0.28 ± 0.13, Fig. 3a2): only one participant showed an average influence higher than 0.5. In line with this egocentric bias, within-subject variability in influence was predicted by the agents’ normalized response distance (B = − 0.421, β = − 0.283, z = − 7.44, *p* < 0.001, Supplementary information, model 2.1), with lower distances predicting higher susceptibility to the partner’s opinion (Fig. 3b2). Participants’ (normalized) estimation error was a good (although weaker) predictor of within-subject variability in influence (Mixed-effect model, B = − 0.310, β = − 0.191, z = − 6.01, *p* < 0.001, Supplementary information, model 2.2). As in Experiment 1, we used the former as control variable in the following sections of the manuscript. Participants’ ratings of own and other’s accuracy were not statistically different (own: 6.19 ± 1.04; partner: 5.78 ± 1.77. Wilcoxon signed-rank test, z = 1.27, r = 0.15, η^2^ = 0.02, *p* = 0.203), although participants’ accuracy was considerably lower than that of the computer algorithm (perceptual error (cm) normalized by stimulus length (cm), participants: 0.21 ± 0.09; partner: 0.10 ± 0.01. Wilcoxon rank-sum test, z = − 7.30, r = 0.86, η^2^ = 0.74, *p* < 0.001). Taken together, these findings reveal that participants over-estimated their own performance compared to that of their partner, as did participants in Experiment 1.

In Experiment 1, we had not found any between-subject relationship between individual average influence and variables expressing the individual perceived reliability of own and partner’s accuracy. On the contrary, in Experiment 2 average influence was significantly predicted by mean normalized distance between the two agents’ estimates (Spearman correlation, rho = − 0.53, *p* < 0.001), mean normalized estimation error (rho = − 0.57, *p* < 0.001), rating of the partner’s performance (rho = 0.58, *p* < 0.001) and difference between ratings of own and partner’s accuracy (rho = − 0.64, *p* < 0.001, Fig. 3c2). All these results were significant at Bonferroni-corrected threshold (*p* = 0.01, n. comparisons = 5). There was no significant effect between average influence and own performance rating (Spearman correlation, rho = − 0.22, *p* = 0.189). These results are consistent with the idea that the influence exerted by the computer partner on participants strictly depended on the participants’ perceived reliability of the partner’s estimates. These findings, in light of the null results of Experiment 1, suggest that susceptibility *towards peers* is not fully explained by informational mechanisms and is indeed affected by inherently social factors.

#### Indirect reciprocity task

In Experiment 2, participants faced the same task described in Experiment 1, but they were aware that perceptual inferences and final decisions were produced by computer algorithms, although their response patterns were entirely unknown. All other aspects of the experimental task were identical to those of Experiment 1.

First, we compared participants’ influence in Susceptible and Unsusceptible conditions. A mixed-effect model revealed no significant effect of condition (Supplementary information, model 2.3). In contrast to Experiment 1, participants tended to anti-reciprocate, rather than reciprocate, the consideration received in the observation turns (Susceptible, 0.31 ± 0.13; Unsusceptible, 0.33 ± 0.14; B = − 0.027, β = − 0.111, z = − 1.71, *p* = 0.088, Fig. 4a2,b2). From an informational perspective, this is consistent with the observed decrease in the perception of own accuracy in the Unsusceptible condition compared to the Susceptible one (Susceptible: 6.56 ± 1.05; Unsusceptible: 5.97 ± 1.21. Wilcoxon signed-rank: z = 3.24, r = 0.38, η^2^ = 0.15, *p* = 0.001), which led participants to rely more on the partner in the decision turns of the Unsusceptible condition. Moreover, participants’ ratings of the partner’s accuracy were higher in the Unsusceptible than in the Susceptible condition (Susceptible: 5.67 ± 1.60; Unsusceptible: 6.14 ± 1.51. Wilcoxon signed-rank: z = − 2.06, r = 0.24, η^2^ = 0.06, *p* = 0.039), although this effect did not survive Bonferroni correction for multiple comparisons (threshold, *p* = 0.025, n. comparisons = 2).

The absence of an effect of reciprocity was corroborated by a Wilcoxon signed-rank test revealing no significant difference in influence across conditions (z = − 1.62, r = 0.19, η^2^ = 0.04, *p* = 0.106) and applied when controlling for the agents’ response distance (Supplementary information, model 2.4).

Eventually, we analyzed differences between the Final block and the preceding main experimental block in terms of influence (Fig. 5a2,b2). In contrast with results of Experiment 1, we did not find any effect of block (Mixed-effect model, B = − 0.009, β = − 0.036, z = − 0.75, *p* = 0.452, Supplementary information, model 2.5; Wilcoxon signed-rank test, z = − 0.86, r = 0.10, η^2^ = 0.01, *p* = 0.387). We found the same null results when controlling for the effect of agents’ response distance (Supplementary information, model 2.6. Taken together, results of the Final block in Experiment 1 and Experiment 2 suggest that social influence *towards peers* is modulated by downstream mechanisms of reciprocity.

## Discussion

In the current study, we tested the hypothesis that social influence in multi-agent systems is modulated by indirect reciprocity. Recent evidence^26^ has shown that individuals *directly* reciprocate the consideration they receive from a partner. Here we hypothesized that reciprocal social influence could also arise *indirectly* in groups in which their members do not have the possibility to interact and share opinions with each of their peers, but have knowledge of their individual level of social susceptibility.

In Experiment 1, we investigated how participants revised perceptual judgment in light of the opinion of an alleged human partner (passive agent, *Social influence task*) and whether participants’ susceptibility towards the same partner could be modulated by the susceptibility expressed by a third alleged peer (active agent) who could revise their own estimates in light of participants’ opinion (*Indirect reciprocity task*). In Experiment 2, a new pool of participants faced the same two tasks, but participants were aware that their partners’ responses were computer-generated.

Results of the *Social influence task* with an alleged human partner (Experiment 1) reveal that between-subject heterogeneity in the influence exerted by the partner was not predicted by participants’ perceived reliability of the partner’s perceptual estimates. On the contrary, when participants knew that the partner was in fact a computer algorithm (Experiment 2), individual differences in influence were strongly predicted by the perceived accuracy of the partner. These results indicate that, in Experiment 1, participants’ susceptibility towards the partner’s opinion was not strictly dependent on the perceived reliability of the partner’s responses. This is in line with previous results in social decision-making revealing the existence of normative biases in the process of integration of the information provided by peers in social scenarios^19,23,24,29,30^. We also underline that, in both experiments, participants’ perception of own and other’s performance was rather inaccurate: in particular, participants relied much more on their own estimate in their final decisions, even if the partner’s accuracy was considerably higher. This finding is consistent with extensive evidence in decision-making and advice taking literatures, showing egocentric biases in the integration of other agents’ feedbacks and opinions^31–37^.

Results of the *Indirect reciprocity task* (Experiment 1) revealed that participants were more influenced by the opinion of a partner (passive agent) when another peer (active agent) has shown high susceptibility (Susceptible condition) rather than low susceptibility (Unsusceptible condition) to participants’ judgments. We have shown that this effect cannot be explained by changes in the perception of own and partner’s reliability: in fact, the passive agent’s accuracy was rated equally in Susceptible and Unsusceptible conditions and, most importantly, participants rated their own accuracy *higher* in the Susceptible than in the Unsusceptible condition. If participants had followed reliability criteria to guide their final decisions, they would have relied more on the partner’s opinion in the Unsusceptible condition. Furthermore, results of Experiment 2 reveal that the effect of reciprocity disappeared when participants knew that they were not interacting with real participants (Experiment 2), suggesting that the reciprocal mechanisms observed in Experiment 1 stem from the social nature of the interactive setting. Crucially, this is the first study to reveal that the process of in-group diffusion and updating of information is regulated by *indirect* reciprocity mechanisms, offering novel insights into how knowledge is shared in multi-agent systems. In particular, our results suggest that the public expression of one’s opinions may be dynamically adjusted based on the current level of susceptibility of the members of one’s social group, highlighting the importance of normative considerations in the process of updating of collective knowledge.

An important question is, therefore, why indirect reciprocity arises in social influence contexts. In traditional cooperative settings, indirect reciprocity can emerge by either *upstream* or *downstream* mechanisms. *Upstream* indirect reciprocity assumes that individuals who have recently received help may feel the urge to help someone else^11–14^. *Downstream* indirect reciprocity predicts that helping others increases the probability of receiving help by modulating reputation^3,5,15–18^. We tested the hypothesis that reciprocity among individuals could be modulated by downstream mechanisms: in particular, we hypothesized that reciprocity is sustained by expectation of future interactions with the same partners, which should elicit mechanisms of reputation control. Therefore, we manipulated participants’ knowledge of future interactions with their partner(s) at the end of the *Indirect reciprocity task*. Results of Experiment 1 indicate a significant drop in the influence exerted by the passive agent when participants did not expect future interactions with the active agent. This effect was absent in Experiment 2, suggesting that part of the susceptibility shown by participants in Experiment 1 was directed to control reputation, with the goal to increase the probability of receiving consideration from the active agent in the near future^26^. This result is reminiscent of classical results in game theory revealing that (reciprocal) cooperation in finitely repeated games, like the Prisoner’s dilemma, declines drastically when players do not expect further interaction with their partner(s)^38–40^, highlighting an important utilitarian component of cooperation^41–43^. In this regard, reciprocal behavior in social influence contexts can be interpreted as behaviorally advantageous if we assume that exerting influence over others is rewarding for individuals. This interpretation is consistent with neuroimaging findings showing activity in the human reward brain network when we exert influence over others^27^ and when others align their opinion with ours^44,45^. Moreover, if our opinions are ignored by others, we may be subject to the typical negative emotional reactions characterizing social exclusion, which has been shown to recruit the same neural network underlying physical pain^28^. In these terms, people may be willing to pay a behavioral cost (by reciprocating influence and therefore not maximizing accuracy) to obtain social fulfilment and avoid social distress^26^.

Nevertheless, we do not claim that the emergence of indirect reciprocity in social influence settings is entirely due to these downstream mechanisms. Conversely, we believe that upstream and downstream processes may interact in supporting reciprocal behavior in these contexts. Further research is needed to understand the interplay between these two dimensions. One possibility would be to manipulate the extent to which the interacting partners receive feedback about others’ behavior. In the current study, all the interacting partners possess full knowledge about the choices of their counterparts, which is fundamental to explore the *downstream* mechanisms of indirect reciprocity. Nonetheless, if participants interacted with agents that cannot see participants’ (final) decisions, they would lose the opportunity to strategically manage reputation to maintain their influence over their partners. In this scenario, the emergence of indirect reciprocity would suggest the additional presence of *upstream* mechanisms modulating reciprocal behavior. Another factor that could drive indirect reciprocal behavior is status competition. In particular, an individual whose opinion is systematically ignored may indirectly reciprocate influence to signal their contrariness to accept an inferior position within the group^46,47^. In this regard, future studies could allow participants to send feedbacks in response to a signal of (un)susceptibility by a partner, or even permit monetary punishment towards unsusceptible agents, in order to specifically characterize individuals’ reactions to others’ (un)susceptibility in social influence settings.

Another fundamental dimension that may be explored in future studies is the informational one. We know that humans naturally tend to ascertain the reliability of socially acquired information before using it to guide their behavior^48,49^. Moreover, they selectively take advice from reliable informants, using information about their confidence^20,50^ and expertise^51–53^. In our study, all these behavioral features were unknown to participants, who could just infer own and others’ accuracy by relying on a very limited set of information. Future studies may overtly provide information about the interacting agents’ expertise, confidence or uncertainty, in order to better understand the interplay between informational and normative consideration in the process of behavioral optimization.

Altogether, our results reveal that our susceptibility towards peers can be modulated by indirect reciprocity mechanisms, shedding new light on the emergence of normative distortions in the processes of information exchange within groups. An interesting question is whether (and in what circumstances) these distortions may be collectively beneficial or detrimental. In this regard, Bayesian theories recommend that individuals weight others’ opinions by their reliability in order to collectively benefit from sharing opinions^54,55^. Nevertheless, reciprocity of social influence may lead, in principle, to positive outcomes whenever single individuals’ knowledge is extremely limited: in these contexts, reciprocity could trigger a collaborative attitude among the members of a social group, who may benefit from gathering their bounded knowledge, leveraging the so-called “wisdom of crowds”^56,57^.

## Methods

### Overview: participants and procedure

Seventy-two participants participated in our study (48 females, mean age: 31.00, SD: 10.35). Half of the participants participated in Experiment 1 (N = 36, 25 females, mean age: 30.17, SD: 10.31), while the other half took part in Experiment 2 (N = 36, 23 females, mean age: 31.83, SD: 10.46). The target sample size was estimated based on the predicted statistical analyses aimed to detect a significant within-subject effect of condition on participants’ social influence in each of the two experiments. The recommended sample size for a Wilcoxon signed-rank test, assuming power of 0.8 and medium effect size (i.e., d = 0.5), is indeed 35. At the same time, a sample size of 34 is recommended for designs with one within-subject two-levels independent variable^58^, always assuming power of 0.8 and medium effect size (i.e., d = 0.5).

In both experiments, all participants completed the entire experimental paradigm, which included two different tasks that were performed in this exact order: *Social Influence task* and *Indirect reciprocity task*. The only difference between the two experiments lies in the participants’ belief about the nature of the partners of the two tasks: human (Experiment 1) or computer (Experiment 2). In fact, the partners’ responses in both experiments were generated by the same computer algorithms, the response patterns of which were unknown to participants. Participants performed every task on a wide touch-screen (43.69 × 24.07 cm). In order to allow participants to respond with high spatial accuracy, we provided a touch-pen with an ultrathin tip. Participants were told that their reimbursement would be calculated based on their performance and, in particular, on the accuracy of both their initial and final estimates in both tasks. The accuracy of the partner(s) was not supposed to have an impact on participants’ outcomes, and vice versa. However, everyone received a fixed amount (15 euros) at the end of the experiment (see paragraph “Cover story and debriefing” for more details). The study was approved by the local ethics committee (Azienda Sanitaria Locale Genovese N.3, protocol: IIT_wHiSPER) and all participants gave informed consent. All experiments were performed in accordance with the relevant guidelines and regulations.

### Experiment 1

#### Experimental design

##### Social influence task

In the *Social influence task* (Fig. 2a,b), participants believed to interact with another participant (passive agent). During the experiment, we referred to this agent as “blue participant” and color-coded the agent’s responses in blue. At the beginning of each trial, participants performed a perceptual inference task in which they had to reproduce the lengths of visual stimuli. Participants saw two consecutive light flashes (red disks of 0.57 cm diameter, duration 200 ms) appearing on a visible horizontal white line crossing the whole screen at its central height. The first disk was positioned at a variable distance from the left border of the screen (0.6–6.6 cm). After its disappearance and an additional inter-stimulus interval of 200 ms, a second disk appeared at a variable distance to its right. The distance between the first and the second disk is defined as the target stimulus length (*s*). Participants were asked to touch a point on the white line, to the right of the second disk, in order to reproduce a segment (connecting the second and the third disk) that matched the target stimulus length. In each trial, the target stimulus length *s* (i.e., distance between the first two disks) was randomly selected from 11 different sample distances (min: 8 cm, max: 16 cm, step: 0.8 cm). Right after the touch of the screen, a third red disk marked with a vertical red line and the word YOU appeared in the selected position. No feedback about the accuracy of the response was provided.

Participants were also told that, during this interval, the very same stimuli would be presented to the passive agent, who would choose a point to reproduce the same distance. After the participant’s estimate, the partner’s simulated estimate would be shown after a jittered time interval (1–1.25 s), marked with the acronym BP (Blue Participant) in blue. However, we stressed that their partner was asked to make their response at the same time as they did. Moreover, participants were told that their estimate would be shown to the other participant *after* their partner’s response.

Right after the appearance of the passive agent’s estimate, participants had the opportunity to make a final decision and potentially revise their initial estimate, choosing any position between their previous response and the other agent’s response, but not outside this range. We computed the *influence* index as the distance (in cm) between the participants’ final and initial choice divided by the distance (in cm) between the two agents’ initial estimates (Fig. 2a). These two distance measures have been collected at single-pixel (sub-millimeter) resolution. In these terms, participants’ influence express the relative weight assigned to the judgment of the two interacting partners. The position of the final decision was marked with a green line and the word “FINAL” in green.

After the final decision was made, the position of the three choices (participant’s and partner’s estimates and participant’s final decision) remained visible on the screen for 1 s. Participants were informed that their final decision would be shown to their partner, who could just observe the participant’s final decision and could not modify their own estimate. The task consisted of 66 trials divided in three blocks by two brief pauses. At the end of the task, participants were asked to evaluate from 1 to 10 their own and the passive agent’s accuracy in terms of perceptual estimate (ignoring the final decision).

The specific parameters of the presented visual stimuli were selected with the aim to ensure high uncertainty in participants’ estimates, to minimize the probability that participants would be highly confident about their response. High confidence would have decreased the need of seeking advice from the partners. Given the difficulty of the perceptual inference task, the *Social influence task* was preceded by 66 individual training trials in which participants had simply to perform the perceptual estimates without receiving any information about their accuracy or the response of another participant. Participants were told that the passive agent also underwent the individual training trials before the *Social influence task*, to guarantee the perception of equal prior experience in the perceptual task.

We told participants that their reimbursement would be calculated based on the accuracy of both their own initial and final responses.

##### Indirect reciprocity task

In the *Indirect reciprocity task* (Fig. 2a,b), participants believed to interact with an additional participant (active agent) along with the same participant with whom they had interacted in the *Social influence task* (passive agent). During the experiment, we referred to the active agent as “yellow participant” and color-coded their responses in yellow to distinguish them from those of the passive agent. The task consisted of two types of alternating turns, which prescribed the partners involved in the interaction and the identity of the agent who would have made the final decision. In decision turns, participants interacted with the passive agent, while in observation turns they played with the active agent.

*Decision turns* were identical to those in the *Social influence task*: the participant made their own perceptual estimate and then observed the response of the passive agent; eventually, *the participant* had to make the final decision, choosing any position between their own perceptual estimate and the passive agent’s one.

In the *observation turns*, the participant made their estimate, then observed the one of the active agent and eventually observed the final decision taken by *the active agent*. The final decision of the active agent was presented after a jittered time interval ranging from 1.25 to 3 s to simulate the typical human variability in decision response times. Participants could not revise their estimate in the observation turns. The active agent’s final decision was manipulated during the task in order to express two different types of behavior: in the Susceptible condition, the agent was highly influenced by the participant’s response, while in the Unsusceptible condition the agent was much less influenced by the participant’s estimate and tended to confirm their own first response (for a detailed description of these algorithms, see paragraph “Alleged partners’ behavior”). Participants were not informed about the existence of these two different conditions. The presentation of Susceptible and Unsusceptible conditions followed a within-subject block design. The order of presentation of the two different blocks was counterbalanced across participants. Each of the two blocks consisted in 66 trials (33 decision turns and 33 observation turns), divided into three sub-blocks by two brief pauses.

During the experiment, we referred to decision and observation turns as *blue* and *yellow* turns, respectively. The estimates of the passive agent and the active agent were color-coded in blue and yellow and highlighted by the acronyms BP and YP, respectively. In both turns, the position of every response was kept visible on the screen for 1 s after the final decision.

Between the two main blocks (Susceptible and Unsusceptible), we introduced a Transition block consisting of 22 trials, in which we implemented a smooth transition in the behavior of the algorithmic agent in order to make the observed behavioral change look more natural and gradual (see paragraph “Transition and Final blocks”), as in Mahmoodi et al.^26^.

At the end of these 154 trials (Susceptible, Transition and Unsusceptible block), we added 22 trials characterized by a different alternation of turns (Final block): first participants faced 11 observation turns in a row, and eventually they underwent 11 decision turns in a row. Participants were informed about this change in the order of turns only at the beginning of the Final block and were told that the experiment would be finished after the 11 decision turns. We introduced the Final block to test whether participants would have decreased their level of influence when they did not expect future interactions with the active agent.

In sum, the task consisted of 176 trials. Participants were told that for the entire length of the task, including the Final block, all three participants could observe the perceptual estimates and the final decisions of every other peer in every type of turn.

During the task, there were pauses every 22 trials. After every pause, participants had to touch the screen to re-start the experimental task and had to wait a jittered interval (0–4 s) in which they could see the following sentence on screen: “Waiting for the other participants…”. In three of these pauses, participants were asked to evaluate from 1 to 10 their own, the passive and the active agents’ accuracy in the perceptual estimates, without considering the final decisions. In addition, they were asked to estimate how much the active agent would value the participant’s accuracy (from 1 to 10). Specifically, participants were asked to rate accuracies once during the Susceptible block (in the last pause of the block, after 44 susceptible trials), once right after the Transition block, and once during the Unsusceptible block (in the last pause of the block, after 44 unsusceptible trials).

As in the *Social Influence task*, participants were told that their reimbursement would be calculated based on the accuracy of both their own initial and final response.

#### Alleged partners’ behavior

##### Perceptual estimate

Passive and active agents’ simulated perceptual estimates were based on the same probabilistic algorithms. The positions of their individual responses were randomly chosen from a gaussian distribution centred at the correct response (SD: 1.52 cm). The standard deviation of the response error distribution was chosen to maintain a balance between variability, credibility and accuracy of response, taking into account the high level of difficulty faced by participants in the perceptual task. In fact, given the considerable participants’ perceptual error in the task, we aimed to control the variance of the algorithm in order to prevent participants from recurrently observing extremely high discrepancies between the two responses. This would have highly affected the perceived reliability of the partners or even their credibility as real participants. Therefore, the selected standard deviation of the response distribution of the algorithm was set to be 25% lower than the observed standard deviation of participants’ responses, as estimated in a pilot study. Nevertheless, we also considered the possibility that few participants could be extremely accurate in the task. In this case, the participant and partner would have selected close responses very often, impeding the observance of variability in participants’ final estimates. For this reason, in half of the trials in which the sampled estimate of the algorithm was rather close to the one of the participant (i.e., d < 0.5 cm), the algorithm re-sampled a new estimate from the distribution (i.e., until d > 0.5 cm).

This response distribution was used both in the *Social Influence* and in the *Indirect Reciprocity task*.

##### Active agent’s final decisions in the indirect reciprocity task

In the *Indirect reciprocity task*, we systematically manipulated the final decisions of the active agent during the observation turns in order to reproduce two types of behaviors characterized by different levels of susceptibility in distinct blocks (Susceptible and Unsusceptible).

In particular, we manipulated the level of influence of the active agent (i.e., shift towards the participant’s estimate in the final decision) which, at each round, could range from 0 to 1: an influence of 1 corresponds to a final decision coinciding with the participant’s perceptual estimate, while an influence of 0 corresponds to a final decision coinciding with the agent’s estimate.

In detail, in each trial of the Susceptible block the active agent’s influence was chosen randomly with a probability of 0.45 from a uniform distribution in the interval[0.75, 1], with a probability of 0.35 from a uniform distribution in the interval[0.5, 0.75], with a probability of 0.15 from a uniform distribution in the interval[0.25, 0.5] and with a probability of 0.05 from a uniform distribution in the interval[0, 0.25].

In the Unsusceptible block, in each trial the active agent’s influence was chosen randomly with a probability of 0.80 from a uniform distribution in the interval[0, 0.25], with a probability of 0.15 randomly from a uniform distribution in the interval[0.25, 0.5] and with a probability of 0.05 randomly from a uniform distribution in the interval[0.5, 0.75].

On average, the influence that the agent took from the participants was 0.64 (between-subject SD = 0.03) and 0.18 (between-subject SD = 0.02) in Susceptible and Unsusceptible conditions, respectively. The average standard deviation in the within-subject distribution of influence was 0.26 and 0.16 in Susceptible and Unsusceptible conditions, respectively.

##### Transition and final blocks

Between the two main blocks of the experiment (Susceptible and Unsusceptible), we introduced a smooth transition in the behavior of the active agent. For the transition from the Susceptible to the Unsusceptible block, the active agent’s influence in the 11 observation turns was chosen twice from a uniform distribution in the interval[0.75, 1], then twice from a uniform distribution in the interval[0.5, 0.75], then three times from a uniform distribution in the interval[0.25, 0.5] and lastly four times from a uniform distribution in the interval[0, 0.25], in this exact order. Conversely, for the transition from the Unsusceptible to the Susceptible block, the amount of influence was chosen from the same distributions, but it was presented in the opposite order, moving sequentially from the interval[0, 0.25] to the interval[0.75, 1].

The Final block included 11 observation turns where the influence that the agent took from the participants followed the same probabilistic distribution of the last condition faced in the *Indirect Reciprocity task*.

### Experiment 2

Experiment 2 was identical to Experiment 1 with one exception: participants did not believe to interact with two other naive participants, but they were aware that, in each turn, their partner’s perceptual estimates and final decisions were, in fact, computer-generated. This was the case for both the *Social influence* and the *Indirect reciprocity tasks*. In both tasks, the patterns of response, both in terms of perceptual inferences and final decisions, were identical to Experiment 1 and unknown to participants. In particular, the *Indirect reciprocity task* included observation turns and decision turns, organized in Susceptible, Transition, Unsusceptible and Final blocks in the same way as in Experiment 1. The order of the two main experimental conditions (Susceptible and Unsusceptible) was counterbalanced across participants. Ratings of own perceptual accuracy, accuracy of the computer partner and second-order belief of the computer concerning participant’s accuracy were collected with the same modalities of Experiment 1 in both tasks.

### Statistical data analysis

Most of the analyses reported in this work focus on analysing variation of the same dependent variable (i.e., influence) as a function of endogenous variables (i.e., distance between agents’ responses, estimation error, performance ratings) and exogenous experimental factors (i.e., experimental conditions). We used mainly mixed-effect models with influence as dependent variable and random effects at the subject level. In all the models of the paper, random effects have been applied: (1) to the intercept, to adjust for the baseline level of influence of each subject and model intra-subject correlation of repeated measurements; (2) to all slopes of the independent variables, to model the inter-subject variability in the relationship between dependent and independent variables. The variance–covariance matrix of all models was estimated using robust variance estimator^59–61^ to obtain heteroscedasticity-robust stand errors clustered at the subject level. Specification and results of each model have been described in detail in the Supplementary information.

In addition to mixed-effect models, throughout the paper we also directly compared individual variables (e.g., average influence, average distance, indirect reciprocity index, performance ratings) across conditions and agents. Since these dependent variables (i.e., individual means) often show some degree of skewness and, in some cases, show a violation of the normality distribution assumption, we used non-parametric tests (Wilcoxon signed-rank test; Wilcoxon rank-sum test) through the entire paper for consistency. All tests are two-tailed and report z statistic, p. value and effect sizes (r, η^2^). For the same reason, we used non-parametric correlation tests (Spearman’s rank correlation). The formulas used for the calculation of the effect sizes can be found in Cohen^62^ and Fritz et al.^63^: r = Z/√N (total number of observations); η^2^ = Z^2^/N.

All analyses of Experiment 1 and Experiment 2 include the entire sample of 72 subjects.

### Cover story and debriefing

Before starting the experiment, participants in Experiment 1 were told that they would be performing the task with other two naive participants, located in two different neighboring experimental rooms. In fact, participants interacted with computer algorithms. At the end of the experiment, we asked participants indirect questions about the experiment to assess whether they have believed that the two partners were two real participants. All participants believed the cover story.

Moreover, participants in both Experiment 1 and Experiment 2 were told that their performance would be affected by their accuracy in terms of perceptual estimation and final decision. Actually, they all received a fixed amount (15 euros) at the end of the experiment, following the guidelines of the Italian Institute of Technology concerning the application of a fair reimbursement for voluntary participation in experimental research. The final debriefing revealed that all participants, during the experiment, believed that their final reimbursement would be affected by their performance.

Eventually, we extensively debriefed participants about the experimental procedures, the reasons underlying the modality of reimbursement and the goals of our research, in accordance with the relevant ethical guidelines.

We acknowledge that the standards on the use of deception vary widely across disciplines and we are aware that this practice may be disputed by researchers in certain fields. We have elaborated more on this issue in a dedicated section of the Supplementary information (Supplementary methods, paragraph “On the use of deception in the current work”), in which we describe the motivations underlying the use of deception in our experimental protocol.

## Supplementary Information

Supplementary Information.

## Data Availability

The datasets supporting all the analyses and the findings included in the current study are publicly available in a dedicated OSF repository at: https://osf.io/cqjb9/

## Code availability

All the scripts used for collecting data and generating results during the current study are available from the corresponding author upon request.
