# Supplementary material for: Dynamic modulation of social influence by indirect reciprocity
Source: Sci Rep. 2021 May 27;11:11104. doi: 10.1038/s41598-021-90656-y (PMC8160268; doi:10.1038/s41598-021-90656-y)
Supplement: Supplementary file 1 — Supplementary Information. [file 41598_2021_90656_MOESM1_ESM.pdf]

Supplementary information

Dynamic modulation of social influence by indirect reciprocity

Joshua Zonca*[1], Anna Folsø [2], Alessandra Sciutti [1]

1. Cognitive Architecture for Collaborative Technologies (CONTACT) Unit, Italian Institute of Technology, Genoa, Italy.

2. Department of Informatics, Bioengineering, Robotics and Systems Engineering, University of Genoa, Genoa, Italy.

***Corresponding author:** Correspondence to Joshua Zonca, Italian Institute of Technology, Via Enrico Melen, 83, 16152 Genova, GE (Italy). Email: joshua.zonca@iit.it

Supplementary results

We report the details of the models ran in the present work (Experiment 1 and Experiment 2). For all models**,** the intercept as well as the coefficients of the independent variables were allowed to vary across participants including random effects at subject level. We estimated the variance-covariance matrix of all models using robust variance estimator to obtain heteroscedasticity-robust stand errors clustered at the subject level.

In every model equation, β expresses coefficients of fixed effects, while u indicates random effects.

In the fixed-effect results, B express unstandardized regression coefficients, while β (in brackets) represents standardized regression coefficients. Independent categorical variables (condition and block, 2 levels) have been treated as dummy variables.

Experiment 1

Social influence task

Model 1.1: Effect of normalized agents' response distance on influence.

We tested the effect of the distance between the participant's estimate and the passive agent's one on trial-by-trial influence. We used the following mixed-effect linear model:

$$I = \beta_0 + \beta_1 d + u_0 + u_1 d + \varepsilon$$

I is the influence of the partner on the participant, and d is the normalized distance between the two agents' responses (response distance (cm) / stimulus length (cm)).

Results:

Influence	B (β)	Robust Std. Err.	z	p	95% Conf. Interval	
Norm. distance	- 0.181 (- 0.145)	0.055	- 3.28	0.001	- 0.289	- 0.073
N. obs	2376					
N. groups	36					

Model 1.2: Effect of normalized estimate error on influence.

We tested the effect of participants' normalized estimate error on participants' trial-by-trial influence. We used the following mixed-effect linear model:

$$I = \beta_0 + \beta_1 e + u_0 + u_1 e + \varepsilon$$

I is the influence of the partner on the participant, and e is the normalized estimate error (estimate error (cm) / stimulus length (cm)).

Results:

Influence	B (β)	Robust Std. Err.	z	p	95% Conf. Interval	
Norm. error	- 0.100 (- 0.075)	0.049	- 2.06	0.039	- 0.196	- 0.005
N. obs	2376					
N. groups	36					

Indirect reciprocity task

<u>Model 1.3: Effect of condition on influence.</u>

We tested the effect of condition (Susceptible or Unsusceptible) on participants' trial-by-trial influence (decision turns). We used the following mixed-effect linear model:

$$I = \beta_0 + \beta_1 c + u_0 + u_1 c + \varepsilon$$

I is the influence of the partner on the participant, and c is the condition factor. The Unsusceptible condition is the reference category in the model output.

Results:

Influence	B (β)	Robust Std. Err.	z	p	95% Conf. Interval	
Condition (Susceptible)	0.029 (- 0.122)	0.011	2.61	0.009	0.007	0.050
N. obs	2376					
N. groups	36					

<u>Model 1.4: Effect of condition on influence adjusting for normalized agents' response distance.</u>

First, it is important to highlight that there were no difference in terms of average normalized distance between conditions (Wilcoxon signed-rank test, z = - 0.45, r = 0.05, η^2 = 0.00, p = 0.65).

We then added the normalized distance variable to the previous model (1.3) to adjust for the variability captured by agents' response distance, using the following mixed-effect linear model:

$$I = \beta_0 + \beta_1 c + \beta_2 d + u_0 + u_1 c + u_2 d + \varepsilon$$

I is the influence of the partner on the participant, *c* is the condition factor and *d* normalized distance. The Unsusceptible condition is the reference category in the model output.

Results:

Influence	B (β)	Robust Std. Err.	z	p	95% Conf. Interval	
Condition (Susceptible)	0.027 (- 0.115)	0.011	2.47	0.013	0.006	0.048
Norm. distance	- 0.220 (- 0.162)	0.042	- 5.22	< .001	- 0.303	- 0.138
N. obs	2376					
N. groups	36					

Model 1.5: Effect of the Final block on influence.

We tested the effect of the Final block, in which participants were aware of the absence of future interactions with the partners, on participants' trial-by-trial influence. We ran the following model:

$$I = \beta_0 + \beta_1 b + u_0 + u_1 b + \varepsilon$$

I is the influence of the partner on the participant and *b* is the block factor. The block of the main task is set as the reference category in the model output.

Results:

Influence	B (β)	Robust Std. Err.	z	p	95% Conf. Interval	
Block (final)	- 0.028 (- 0.119)	0.011	- 2.52	0.012	- 0.050	- 0.006
N. obs	1584					
N. groups	36					

Model 1.6: Effect of the Final block on influence, adjusting for normalized agents' response distance.

First, we highlight that there were no difference in terms of average normalized distance between the two blocks (main experimental block and final block) (Wilcoxon signed-rank test, z = - 0.27, r = 0.03, $\eta^2 = 0.00$, p = 0.789).

Then we ran a new mixed-effect model adding the normalized distance variable to the previous model (1.5). This is the model equation:

$$I = \beta_0 + \beta_1 b + \beta_2 d + u_0 + u_1 b + u_2 d + \varepsilon$$

I is the influence of the partner on the participant, *b* is the block factor and *d* normalized distance.

Results:

Influence	B (β)	Robust Std. Err.	z	p	95% Conf. Interval	
Block (final)	- 0.028 (- 0.117)	0.011	- 2.56	0.010	- 0.049	- 0.007
Norm. distance	- 0.224 (- 0.163)	0.040	- 5.55	< .001	- 0.303	- 0.145
N. obs	1584					
N. groups	36					

Experiment 2

Social influence task

<u>Model 2.1: Effect of normalized agents' response distance on influence.</u>

We tested the effect of the distance between the estimates of the participant and the partner on participants' trial-by-trial influence. We used the following mixed-effect linear model:

$$I = \beta_0 + \beta_1 d + u_0 + u_1 d + \varepsilon$$

I is the influence of the partner on the participant, and d is the normalized distance between the two agents' responses (response distance (cm) / stimulus length (cm)).

Results:

Influence	B (β)	Robust Std. Err.	z	p	95% Conf. Interval	
Norm. distance	- 0.421 (- 0.283)	0.057	- 7.44	< 0.001	- 0.532	- 0.310
N. obs	2376					
N. groups	36					

<u>Model 2.2: Effect of normalized estimate error on influence.</u>

We tested the effect of participants' normalized estimate error on participants' trial-by-trial influence. We used the following mixed-effect linear model:

$$I = \beta_0 + \beta_1 e + u_0 + u_1 e + \varepsilon$$

I is the influence of the partner on the participant, and e is the normalized estimate error (estimate error (cm) / stimulus length (cm)).

Results:

Influence	B (β)	Robust Std. Err.	z	p	95% Conf. Interval	
Norm. error	- 0.310 (- 0.075)	0.052	- 6.01	< 0.001	- 0.411	- 0.209
N. obs	2376					
N. groups	36					

Indirect reciprocity task

Model 2.3: Effect of condition on influence.

We tested the effect of condition (Susceptible or Unsusceptible) on participants' trial-by-trial influence (decision turns). We used the following mixed-effect linear model:

$$I = \beta_0 + \beta_1 c + u_0 + u_1 c + \varepsilon$$

I is the influence of the partner on the participant, and c is the condition factor. The Unsusceptible condition is the reference category in the model output.

Results:

Influence	B (β)	Robust Std. Err.	z	p	95% Conf. Interval	
Condition (Susceptible)	- 0.027 (- 0.111)	0.016	- 1.71	0.088	- 0.057	0.004
N. obs	2376					
N. groups	36					

Model 2.4: Effect of condition on influence adjusting for normalized agents' response distance.

First, it is important to highlight that there were no difference in terms of average normalized distance between conditions (Wilcoxon signed-rank test, z = - 0.45, r = 0.05, $\eta^2 = 0.00$, p = 0.65).

We then added the normalized distance variable to the previous model (2.3) to adjust for the variability captured by agents' response distance, using the following mixed-effect linear model:

$$I = \beta_0 + \beta_1 c + \beta_2 d + u_0 + u_1 c + u_2 d + \varepsilon$$

I is the influence of the partner on the participant, *c* is the condition factor and *d* normalized distance. The Unsusceptible condition is the reference category in the model output.

Results:

Influence	B (β)	Robust Std. Err.	z	p	95% Conf. Interval	
Condition (Susceptible)	- 0.023 (- 0.096)	0.016	- 1.47	0.141	- 0.054	0.008
Norm. distance	- 0.335 (- 0.215)	0.049	- 6.79	< .001	- 0.432	- 0.238
N. obs	2376					
N. groups	36					

<u>Model 2.5: Effect of the Final block on influence.</u>

We tested the effect of the final block, in which participants were aware of the absence of future interactions with the partners, on participants' trial-by-trial influence. We ran the following model:

$$I = \beta_0 + \beta_1 b + u_0 + u_1 b + \varepsilon$$

I is the influence of the partner on the participant and *b* is the block factor. The block of the main task is the reference category in the model output.

<u>Results:</u>

Influence	B (β)	Robust Std. Err.	z	p	95% Conf. Interval	
Block (final)	- 0.009 (- 0.036)	0.012	- 0.75	0.452	- 0.032	0.014
N. obs	1584					
N. groups	36					

<u>Model 2.6: Effect of the Final block on influence, adjusting for normalized agents' response distance.</u>

First, we highlight that there were no difference in terms of average normalized distance between the two blocks (main experimental block and final block) (Wilcoxon signed-rank test, z = - 0.27, r = 0.03, η^2 = 0.00, p = 0.789).

Then we ran a new mixed-effect model adding the normalized distance variable to the previous model (2.5). This is the model equation:

$$I = \beta_0 + \beta_1 b + \beta_2 d + + u_0 + u_1 b + u_2 d + \varepsilon$$

I is the influence of the partner on the participant, *b* is the block factor and *d* normalized distance.

Results:

Influence	B (β)	Robust Std. Err.	z	p	95% Conf. Interval	
Block (final)	- 0.013 (- 0.052)	0.012	- 1.03	0.305	- 0.037	0.012
Norm. distance	- 0.309 (- 0.199)	0.059	- 5.26	< .001	- 0.425	- 0.194
N. obs	1584					
N. groups	36					

Results of the Final block reveal that participants in Experiment 2 were not affected by expectations of future interactions with their partner, in contrast with participants in Experiment 1. This suggests that participants in Experiment 1 did modulate their susceptibility towards others' judgments in the main experimental blocks of the *Indirect Reciprocity task* following downstream mechanisms of reciprocity. This interpretation is corroborated by evidence showing that participants' final decisions in the Final block, when reputation-based concerns were ruled out, were comparable across experiments (Wilcoxon rank-sum test, $z = 1.38$, $r = 0.16$, $\eta^2 = 0.03$, $p = 0.166$).

Supplementary Methods

On the use of deception in the current work

We acknowledge that the use of deception may be disputed in some research fields. Therefore, we would like to explain the motivations underlying the use of deception in our work.

In the current experimental protocol, participants were told that their final monetary reimbursement would depend on their accuracy, taking into account both perceptual estimates and final decisions. However, they all received a fixed amount (15 euros) at the end of the experiment. Moreover, participants in Experiment 1 believed to interact with two other naive participants, who were supposed to perform the task in two different neighboring experimental rooms. In reality, participants interacted with computer algorithms.

Concerning participants' reimbursement, we highlight that our Ethical committee has been encouraging researchers to intend participants' compensation as a *fair reimbursement* for voluntary participation in experimental research, which should not be affected by participants' individual characteristics and skills. For this reason, the use of incentive-based compensation was not allowed for our research. In this context, the use of deception concerning the final payment was the only way to balance the scientific need for incentivization and the ethical requirements of the Ethical committee.

Concerning the second kind of deception used in our paper (i.e., the use of alleged human participants), we believe that none of our research questions could be investigated properly using real participants. First,

partners' decisions had to be experimentally controlled to allow comparisons across subjects, experimental conditions and between-subject experiments. Second, and most importantly, we have shown that participants' susceptibility to their partner's judgments was modulated by characteristics of the partner's perceptual estimates. By allowing variability in the partner's perceptual responses, we would have introduced an additional, uncontrollable confound in the characterization and interpretation of participants' reciprocal behavior.

We underline that the typical downsides of deception (e.g., lack of trust in the experimenter) are unlikely to have a decisive impact on the current research, nor on future research within the Italian Institute of Technology, where social decision making research involving incentive-based compensation or alleged human partners is very uncommon.

Furthermore, we highlight that participants were extensively debriefed about the experimental procedures, the reasons underlying the modality of reimbursement and the goals of our research, in accordance with the relevant ethical guidelines.

We acknowledge that these practices are generally accepted in fields such as psychology, cognitive science and neuroscience. We also want to emphasize that our work has been inspired by a recent paper [1] that investigates the same topic in the context of *direct* reciprocity of social influence, using a similar experimental design and the involving the same two kinds of deception used in our experimental protocol.

References

1. Mahmoodi, A., Bahrami, B. & Mehring, C. Reciprocity of social influence. *Nat. Commun.* **9,** 1-9 (2018)
